# Asperentin B, a New Inhibitor of the Protein Tyrosine Phosphatase 1B

**DOI:** 10.3390/md15060191

**Published:** 2017-06-21

**Authors:** Jutta Wiese, Hülya Aldemir, Rolf Schmaljohann, Tobias A. M. Gulder, Johannes F. Imhoff

**Affiliations:** 1 GEOMAR Helmholtz Center for Ocean Research Kiel, RD3 Marine Microbiology, Düsternbrooker Weg 20, 24105 Kiel, Germany; jwiese@geomar.de (J.W.); rschmaljohann@geomar.de (R.S.); 2 Department of Chemistry and Center for Integrated Protein Science Munich (CIPSM), Biosystems Chemistry, Technical University of Munich, Lichtenbergstraße 4, 85747 Garching, Germany; h.aldemir@tum.de

**Keywords:** PTP1B, cladosporin, asperentin, *Aspergillus*, deep-sea

## Abstract

In the frame of studies on secondary metabolites produced by fungi from deep-sea environments we have investigated inhibitors of enzymes playing key roles in signaling cascades of biochemical pathways relevant for the treatment of diseases. Here we report on a new inhibitor of the human protein tyrosine phosphatase 1B (PTP1B), a target in the signaling pathway of insulin. A new asperentin analog is produced by an *Aspergillus* *sydowii* strain isolated from the sediment of the deep Mediterranean Sea. Asperentin B (**1**) contains an additional phenolic hydroxy function at C-6 and exhibits an IC_50_ value against PTP1B of 2 μM in vitro, which is six times stronger than the positive control, suramin. Interestingly, asperentin (**2**) did not show any inhibition of this enzymatic activity. Asperentin B (**1**) is discussed as possible therapeutic agents for type 2 diabetes and sleeping sickness.

## 1. Introduction

Fungi are widely distributed in marine environments. It becomes more and more evident, that fungi also occur in extreme marine habitats such as the deep sea. A culture-independent approach showed for the first time the fungal diversity in deep-sea hydrothermal ecosystems in the East Pacific Rise and Mid-Atlantic Ridge [1]. More than 7000 sequences of the small subunit rRNA (18S) were obtained affiliating to 20 phylotypes of the fungal phyla *Chytridiomycota*, *Ascomycota*, and *Basidiomycota*, including a new lineage of the *Chytridiomycota* [1]. More recently 48 phylotypes belonging to *Ascomycota* (*Eurotiomycetes*, *Saccharomycetes*, *Dothideomycetes*, *Sordariomycetes*, *Saccharomycetes*, *Leotiomycestes*) and *Basidiomycota* (*Agaricomycetes*, *Exobasidiomycetes*, *Tremellomycetes*) were detected in the deep-sea sediment of the Pacific Ocean by applying sequencing of approximately 2000 internal transcribed spacer regions (ITS) of fungal rRNA genes. Members of *Fusarium*, a genus of the *Sordariomycetes*, were dominant, but also *Aspergillus* and *Penicillium*, representatives of the *Eurotiomycetes*, were observed [2].

Our culture-dependent studies on the deep Mediterranean Sea revealed a dominance of *Aspergillus* and *Penicillium* in sediment samples representing almost half of all 43 isolates [3]. This finding is also reflected by the dominance of genera of producers of biologically active metabolites from the deep sea as reviewed by Wang et al. [4]. *Aspergillus sydowii* obtained from a deep-sea sample (1000 m) produced 2,3,5-trimethyl-6-(3-oxobutan-2-yl)-4*H*-pyran-4-one and (2*R*)-2,3-dihydro-7-hydroxy-6,8-dimethyl-2-[(*E)*-prop-1-enyl]chromen-4-one, which both exhibit cytotoxic effects on the leukemia cell line P388 [5]. Cytotoxic activities were also shown for oxisterigmatocycstin A–C, metabolites isolated from *Aspergillus versicolor* strain CXCTD-06-6a which was derived from the Pacific Ocean at (600 m) [6]. The same strain also produced diketopiperazine brevianamide W, showing antioxidant activity [7].

In the frame of our studies, to characterize new inhibitors of enzymes playing important roles in signaling pathways relevant for the treatment of diseases, fungi from different marine environments were investigated. The present study describes the production of a new asperentin derivative by an *Aspergillus sydowii* isolate obtained from sediment of the deep Mediterranean Sea (2769 m). This compound strongly inhibited the enzyme protein tyrosine phosphatase 1B (PTP1B), an important target for the treatment of type 2 diabetes [8]. Because this disease belongs to the 10 leading causes of death and has strong negative effects on life quality [9], the discovery of new drugs for the treatment of diabetes mellitus is a great challenge and the results are discussed in this respect.

## 2. Results

### 2.1. Origin and Classification of the Producer Strain LF660

The origin and classification of the strain LF660 was described recently [10]. Briefly, LF660 was obtained from a deep-sea sediment sample taken from the Levantine Basin SE of Crete (Mediterranean Sea) in 2769 m water depth. The fungus was classified phylogenetically using the sequence of the ITS1-5.8S rRNA-ITS2 gene fragment, to be a member of the genus *Aspergillus*. It showed very close relationships to *Aspergillus sydowii* strain EN50 (GenBank accession number FJ807779) and *Aspergillus versicolor* strain DY20.1.1 (GenBank accession number LC105698), exhibiting sequence similarities of 100% and 99.8%, respectively. Because the sequence is identical to that of *Aspergillus sydowii*, we consider the isolate as a strain of this species, though its identification is not unambiguous. The ITS1-5.8S rRNA-ITS2 gene fragment sequence of strain LF660 was deposited under the GenBank accession number KX688043.

### 2.2. Isolation and Structure Elucidation of Asperentin B *(**1**)* by Aspergillus sydowii LF660

**Figure 1:**
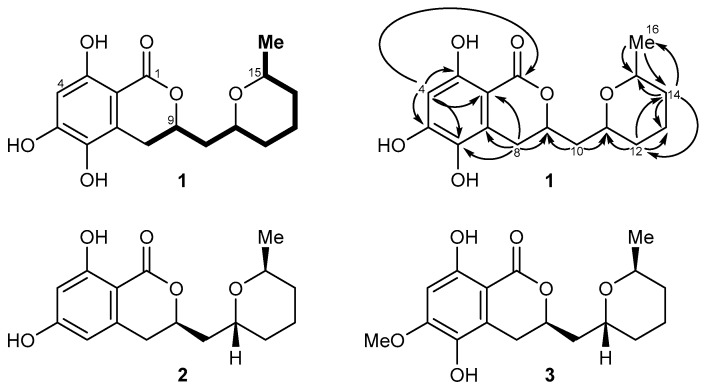
Top: structure and NMR data of asperentin B (**1**): selected ^1^H-^1^H-COSY (bold lines) and HMBC correlations (H → C) are shown. Bottom: structures of the parent compound asperentin (**2**, also called cladosporin) and the known methyl ether **3**.

The HPLC-MS analysis of the crude extract of the fermentation broth revealed 10 peaks. *Aspergillus sydowii* LF660 was recently shown to be the producer of the benzoic acid derivative sydonic acid, diketopiperazine alkaloid rugulosuvine, and benzocoumarin pannorin, an inhibitor of the glycogen synthase kinase GSK-3 β [10]. In the course of the current work, we isolated and purified an additional metabolite from the fermentation broth of this strain. This metabolite was selected for structure elucidation because ^1^H NMR data indicated the presence of a new asperentin derivative. The molecular composition of C_16_H_20_O_6_ was deduced from HRESIMS data that showed the [M − H]^−^ peak at *m*/*z* 307.1188 (calcd. for C_16_H_20_O_6_, 307.1187), thus including seven double bond equivalents. The UV spectrum of **1** showed maxima at 366, 325, 271, 232, and 214 nm (ε 16071, 17762, 13940, 9119, 1071, ACN/H_2_O = 1/1), indicating a substituted aromatic system. The ^1^H NMR spectrum of **1** contained only a single aromatic proton with a low chemical shift at 6.26 ppm (H-4), showing the aromatic portion to be highly electron rich (Table 1). In addition, three signals of aliphatic CH groups attached to an oxygen function (H-9, H-11, H-15), of five diastereotopic CH_2_ groups (H-8a/b, H-10a/b, H-12a/b, H-13, H-14a/b), and of a methyl unit (H-16) were visible. ^1^H-^1^H-COSY correlations allowed the assembly of a contiguous spin system in the eastern molecular portion of **1** ranging from H-8 to H-16, the connectivity of which was further corroborated by HMBC correlations (Figure 1, top). Further HMBC correlations—in particular, from H-8 to C-2, C-6, C-7, and C-9 as well as from H-4 to C-1, C-2, C-3, C-5, and C-6—allowed the complete assembly of the overall structure of the metabolite to give asperentin B (**1**).

**Figure 2:**
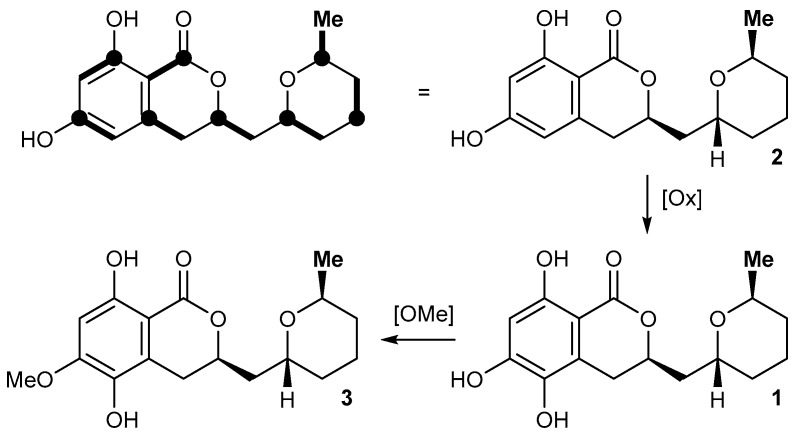
Proposed biosynthetic relationship of the asperentin derivatives **1**–**3**. The bold lines in the top left structure indicate the location of individual C_2_ building blocks from PKS biosynthesis with bold circles depicting former positions of their respective carboxylate function.

Literature search revealed that **1** is a close structural analog of asperentin (**2**, also known as (-)-cladosporin) [11] and compound **3** [12], its 6-dehydroxy- and 5-*O*-methyl analogs, respectively. Comparison of the NMR data of the new metabolite **1** reported here with the published data of **2** and **3** nicely correlated, thus unambiguously validating the proposed structure of asperentin B (**1**). We furthermore found that asperentin (**2**) was indeed likewise produced by strain LF660, as well as by a number of other fungal strains within our strain collection. In all cases, the spectroscopic data of the isolated **2** was fully identical with that of **2** reported in the literature [11]. The optical rotation values of **2** and **3** possessing the stereostructures shown in Figure 1 have negative signs [11,12]. This also holds true for **1**, with ${\left[\alpha \right]}_{D}^{25}$ −17.5 (*c* = 0.11, MeOH). This suggested an identical absolute configuration of the newly isolated **1**, which is also in agreement with the most likely biosynthetic relationship of the asperentins, were **2** would be derived from a polyketide synthase (PKS) precursor, hydroxylated at the aromatic portion to give **1**, followed by *O*-methylation to yield **3** (Figure 2). Consequently, the stereochemistry of **1** was assigned as previously deduced for **2 [12]**.

### 2.3. Biological Activities

Asperentin B (**1**) inhibited the activity of the protein-tyrosine-phosphatase 1B (PTP1B) with an IC_50_ value of 2.05 (±0.15) μM. The positive control used in this bioassay, suramin, exhibited an IC_50_ value of 11.85 (±0.25) μM in our study with the substrate IR5, which accords very well with the IC_50_ value determined by McCain et al. of 11 (±1) μM using *p*-nitrophenyl phosphate as substrate [13]. This corresponds to a stronger effect of **1** by a factor of 6 in comparison to the effect of suramin. Asperentin B (**1**) was activity was poor against the causative agents of acne vulgaris *Propionibacterim acnes* showing an inhibition of 57% at 100 μM test concentration. No antibiotic activity, even at high concentrations of 100 μM in the assays, was observed against *Xanthomonas campestris*, the plant pathogen causing bacterial leaf spot on tomatoes, the wheat plant pathogenic fungus *Septoria tritici*, the yeast *Candida albicans*, as well as against the Gram-positive bacteria *Bacillus subtilis* and *Staphylococcus lentus*. In addition, compound **1** was tested for cytotoxic effects against cancer cell lines using HepG2 and HT29. No activities were not found at test concentrations up to 50 μM. Taking into consideration the lack of antibacterial activity and of cytotoxic effects of asperentin B (**1**), its promising potential as PTP1B inhibitor should be further investigated in future studies.

In contrast to asperentin B (**1**), asperentin (**2**) did not show any activity against PTP1B and *P. acnes*. Both **1** and **2**, did not exhibit cytotoxic activities against the cell line HT29 as well as *X. campestris*, *B. subtilis*, and *S. lentus. S. tritici* and *T. mentagrophytes* were inhibited by asperentin (**2**) at 83% and 100% in comparison to the positive control clotrimazole, respectively, using a concentration of 100 μM. Taking into account the IC_50_ value of 0.18 μM (±0.007) of clotrimazole, the antifungal activity of asperentin (**2**) is very weak.

## 3. Discussion

Several bioactivities have been reported from asperentin (**2**) and derivatives thereof in other studies [11,12,14,15]. Asperentin (**2**) inhibited the growth of five dermatophytes, including *Trichophyton rubrum*, and of the plant pathogen *Rhizoctonia solani* with a minimum inhibitory concentration (MIC) of 75 μg/mL [11]. Monoacetyl asperentin exhibited a MIC of 75 μg/mL against four dermatophytes, including *Trichophyton rubrum*, and against the bacterium *Micrococcus flavus*. The antifungal activity of **2** against eight yeast strains and two filamentous fungi applying a test concentration of 120–150 μg/filter disk was observed by Anke & Zähner [14]. *B. subtilis* was not inhibited as shown in our study. The effects of **2** against the bacteria *Arthrobacter citreus*, *Bacillus brevis*, and *Sarcina lutea* with MIC values of 1 μg/mL, 0.05 μg/mL, and 2.0 mg/mL, respectively, were stronger than those of dimethyl asperentin showing MIC values of >100 μg/mL, 10 μg/mL, and >100 μg/mL, respectively. Jacyno et al. reported, that isoasperentin was slightly more active in the etiolated wheat coleoptile assay than asperentin (**2**). Using a concentration of 1 mM, 100% growth inhibition was observed for isoasperentin in comparison to 81% for **2** [15]. Asperentin (**2**) was the only compound being active against the crop pathogens *Colletotrichum gleosporoides* and *Botrytis cinerea* in comparison to three asperentin derivatives and five analogues thereof [12]. Inhibition of protein tyrosine phosphatase 1B (PTP1B) has not been reported for **2** or any derivatives thereof.

PTP1B is involved in central signaling pathways. It plays a key role in the signaling cascade of insulin and therefore is a key target in the treatment of type 2 diabetes. Drugs for the treatment of diabetes have great economic value. It is a chronic disease of increasing importance worldwide and occurs either when the pancreas does not produce enough insulin or when the body cannot effectively use the insulin. In 2012, diabetes was the direct cause of 1.5 million deaths and high blood glucose was the cause of another 2.2 million deaths. In 2014, 8.5% of adults aged 18 years and older had diabetes. Type 2 diabetes comprises the majority of people with diabetes around the world [16,17]. Resistance to insulin is a predominant pathophysiological factor of this disease. Despite normal insulin levels the glucose uptake from the blood into the cells is reduced [8,16]. PTP1B dephosphorylates the activated insulin receptor (IR) and the insulin receptor substrate and thereby inactivates glucose uptake. Inhibitors of PTP1B can reduce the resistance to insulin and due to enhanced insulin signaling increase the insulin-stimulated glucose uptake. Therefore, PTP1B inhibitors could be used as pharmaceuticals for the treatment of type 2 diabetes and obesity [8,18,19,20]. Metabolites belonging to a broad spectrum of compound classes—including terpenoids, phenolic compounds, alkaloids, as well as semi-synthetic compounds and derivatives thereof, i.e., kinsensoide, lobaric acid, and oleanolic acid—have been patented as promising PTP1B inhibitors [21]. The IC_50_ values for the natural PTP1B inhibitors mainly ranged between 1.5 μM and 30 μM. The semi-synthetic inhibitors were more effective with IC_50_ values mostly in the range of 0.1 μM to 12 μM. In addition, canthinone alkaloids exhibited IC_50_ values in the range of 20 μM to 29 μM and (R)-4-Hydroxy-5-(hydroxymethyl)-3-(1-oxohexadecyl)-2(5H)-furanone (RK-682) inhibited the PTP1B with an IC_50_ value of 4.5 μM [20]. In this study, an IC_50_ value of 2 μM was observed for asperentin B (**1**).

In addition, PTP1B acts as a key player in the development of cancer and in inflammation processes [19]. PTP1B inhibitors are also used for treatment of the sleeping sickness caused by *Trypanosoma brucei* and suramin, which was used as a reference in the bioassay of this study with human PTP1B, is a front-line drug against African trypanosomiasis. As human and trypanosome tyrosine phosphatases show conservation of important functional motifs, specific compounds being active against the human PTP1B quite likely target the enzyme of *T. brucei* [22].

## 4. Materials and Methods

### 4.1. Isolation, Cultivation, Storage, and Classification of the Producer Strain LF660

Strain LF660 was cultivated on WSP30 agar, a modified Wickerham-medium consisting of 1% glucose, 0.5% peptone, 0.3% yeast extract, 0.3% malt extract, 3% sodium chloride (pH = 6.8) [23]. The strain was cryopreserved in liquid nitrogen and in the Microbank System at −80 °C (MAST DIAGNOSTIKA, Reinfeld, Germany). For the genetic characterization of the fungi the ITS1-5.8S rRNA-ITS2 gene fragments were analyzed. DNA-extraction and PCR were performed according to Wiese et al. [24]. Closest relatives were identified by sequence comparison with the NCBI Genbank database using BLAST (Basic Local Alignment Search Tool) [25]. Sequence similarity values were determined with the “bl2seq” tool of the NCBI database [26]. The ITS1-5.8S rRNA-ITS2 gene sequence was deposited under the GenBank accession no. KX688043.

### 4.2. Fermentation and Production of Extracts for the Purification of Compound ***1***

Strain LF660 was inoculated onto agar-plates containing WSP30 medium. After incubation for 35 days at 28 °C, the pre-culture was used for inoculation of 2-L Erlenmeyer flasks containing 750 mL WSP30 medium. The flasks were incubated for 17 days at 28 °C as static cultures in the dark. The mycelium was separated from the culture medium and 7.5 L fermentation broth was extracted using 6.6 L ethyl acetate. The ethyl acetate extract of strain LF660 was evaporated to remove the solvent and re-dissolved in 5 mL methanol. The methanolic solution was subjected to further purification by preparative HPLC.

### 4.3. Isolation of Compound ***1***

Analytical reversed phase HPLC-DAD(UV)-MS experiments were performed using a C_18_ column (Phenomenex Onyx Monolithic C_18_, 100 × 3.00 mm, Phenomenex Inc., Aschaffenburg, Germany) and applying a H_2_O/acetonitrile (ACN) gradient with 0.1% formic acid added to both solvents (gradient: 0 min 5% ACN, 4 min 60% ACN, 6 min 100% ACN; flow 2 mL/min) on a VWR Hitachi Elite LaChrom system (VWR, Darmstadt, Germany) with an L-2450 diode array detector, an L-2130 pump, and an L-2200 autosampler. This HPLC system was coupled to an ESI-ion trap detector with positive ionization (Esquire 4000, Bruker Daltonics, Bremen, Germany) for mass detection.

The preparative HPLC of the crude extract (5 mL) was conducted with a VWR HPLC-UV system (VWR International LaPrep, VWR) equipped with a pump P110, an UV detector P311, a Smartline 3900 autosampler (Knauer, Berlin, Germany), a LABOCOL Vario-2000 fraction collector (LABOMATIC) and a Phenomenex Gemini-NX column (C18, 10 μ, 110 A, 100 × 50 mm, Phenomenex Inc.). An H_2_O/acetonitrile (ACN) gradient with 0.1% formic acid added to both solvents was applied (gradient: 0 min 10% ACN with a flow of 40 mL/min; 0.5 min 10% ACN, 17 min 60% ACN, 22 min 100% ACN, 26 min 10% ACN; flow 100 mL/min). Under these conditions, compound **1** eluted after 12.8 min. From 0.2 g of the raw material, 2.5 mg of compound **1** were isolated as solid.

### 4.4. Structure Elucidation of Compound ***1***

NMR spectra were recorded at 25 °C on a Bruker AVANCE DMX 500 NMR and a Bruker AV 600 (Bruker Daltonics, Bremen, Germany) spectrometer. The compound was dissolved in methanol-*d*_4_. The signals of the residual solvent protons and the solvent carbons were used as internal references (δ_H_ = 3.31 ppm and δ_C_ = 49.0 ppm for methanol-*d*_4_). Masses were acquired using an ESI-ion trap detector with positive ionization (Esquire 4000, Bruker Daltonics, Bremen, Germany). 

### 4.5. Biological Activities Assays

The effect of **1** and **2** on human recombinant protein tyrosine phosphatase 1B (PTP1B) was tested in microtiter plates. The compounds were solved in PTP1B assay buffer containing 100 mM HEPES buffer (pH 7.2), 2 mM EDTA, 2 mM DTT, and 0.1% nonylphenylpolyethylene glycol (NP-40; Biomol, Hamburg, Germany; CatNo. KI-131) in a volume of 45 μL per well. After warming for 10 min to the assay temperature of 30 °C 5 μL of human recombinant PTP1B (Biomol; CatNo. SE332-0050) stock solution (1 ng/μL) were added. The reaction was started with 50 μL of the substrate IR5 (insulin receptor amino acids 1142–1153 with pTyr at position 1146 (Biosyntan, Berlin, Germany) dissolved in PTP1B assay buffer to a concentration of 0.15 mM. The phosphopeptid IR5 comprises the amino acid sequence Thr-Arg-Asp-Ile-pTyr-Glu-Thr-Asp-Tyr-Tyr-Arg-Lys-COOH and is part of the autophosphorylation domain of the insulin receptor (IR). After an incubation period of 25 min at 30°C the reaction was stopped by adding 150 μL of Biomol green (Biomol, CatNo. AK111-0250). PTP1B dephosphorylates the IR5 substrate resulting in the release of orthophosphate which was quantified after incubating for 20 min at 620 nm using the microtiter plate reader Infinite M200 (Tecan Group Ltd., Männedorf, Switzerland). Suramin (Enzo, Lörrach, Germany) was used as positive control. IC_50_ values were determined as duplicates.

Antimicrobial activities of compounds **1** and **2** against *Bacillus subtilis* DSM 347, *Staphylococcus lentus* DSM 6672, *Propionibacterium acnes*, *Xanthomonas campestris*, and *Septoria tritici*, as well as the cytotoxic activity against the cell lines HepG3 (hepatocellular carcinoma) and HT29 (colon adenocarcinoma) were assayed as described by Schneemann et al. [27]. The effect on the growth of human pathogenic yeast, *Candida albicans* DSM 1386, was measured according to Ohlendorf et al. [28]. The activity of compound **2** against the dermatophyte *Trichophyton mentagrophytes* was tested as described by Nagel et al. [29].

## 5. Conclusions

The role of PTP1B in central metabolic pathways relevant for the development of several diseases makes it a key target for the development of new drugs. The inhibition of PTP1B by asperentin B (**1**) with an IC_50_ value of 2 μM makes this compound interesting as a potential candidate for drug development. Its activity is clearly surpassing that of the established drug, suramin. While a low IC_50_ value against PTP1B is an encouraging indicator of potential use of **1** as a therapeutic agent, selective activity against PTP enzymes is also critical. Further studies directed at this issue as well as on the characterization of the binding site and on structure–activity relationships will provide information on the interaction with the enzyme and chemical modifications might improve the efficacy and specificity of asperentin B (**1**). Therefore, asperentin B (**1**) should be considered in further evaluation of possible drug candidates for the treatment of type 2 diabetes, cancer, inflammatory processes, and sleeping sickness.

## Figures and Tables

**Table 1:** NMR data of **1** recorded in MeOH-*d*_4_ on Bruker DMX 500 and AV 600 spectrometers. * HMBC signals not unambiguously assignable due to signal overlap of H-13 and H-14b.

Signal	^1^H	I, Mult, *J*	^13^C	COSY	HMBC
1	-		171.9		
2	-		100.1		
3	-		158.1		
4	6.26	1H, s	101.9	-	1, 2, 3, 5, 6
5	-		155.9		
6	-		135.9		
7	-		126.0		
8a	3.18	1H, dd, 16.8, 3.4	28.6	8b, 9	2, 6, 7
8b	2.66	1H, dd, 16.8, 11.3	28.6	8a, 9	2, 6, 7, 9
9	4.62	1H, dddd, 11.3, 9.1, 3.4, 3.4	77.7	8a/b, 10a/b	-
10a	2.15	1H, ddd, 14.7, 10.4, 3.4	39.4	9, 10b, 11	11
10b	1.80	1H, ddd, 14.7, 9.1, 3.4	39.4	9, 10a, 11	9
11	4.15	1H, m	68.4	10a/b, 12a/b	-
12a	1.72	1H, m	31.5	11, 12b, 13	11, 13
12b	1.41	1H, m	31.5	11, 12a, 13	11, 13, 14
13	1.70	2H, m	19.3	12, 14	*
14a	1.70	1H, m	32.8	13, 15	*
14b	1.33	1H, m	32.8	13, 15	12, 13, 15, 16
15	3.92	1H, m	68.4	16	13
16	1.19	3H, d, 6.5	20.0	15	14, 15

## Supplementary Materials

The following are available online at http://www.mdpi.com/1660-3397/15/6/191/s1.
