# Supplementary material for: Asperentin B, a New Inhibitor of the Protein Tyrosine Phosphatase 1B
Source: Mar Drugs. 2017 Jun 21;15(6):191. doi: 10.3390/md15060191 (PMC5484141; doi:10.3390/md15060191)

Asperentin B, a new inhibitor of the protein tyrosine phosphatase 1B

Jutta Wiese[1], Rolf Schmaljohann[1], Tobias A.M. Gulder[2,*], and Johannes F. Imhoff[1,*]

[1] GEOMAR Helmholtz Center for Ocean Research Kiel, RD3 Marine Microbiology, Düsternbrooker Weg 20, 24105 Kiel, Germany; jwiese@geomar.de (J.W.); rschmaljohann@geomar.de (R.S.), jimhoff@geomar.de (J.F.I.)

[2] Technical University of Munich, Biosystems Chemistry, Department of Chemistry and Center for Integrated Protein Science Munich (CISPM), Lichtenbergstraße 4, 85747 Garching, Germany; **tobias.gulder@**ch.tum.de (T.A.M.G.)

* Correspondence: tobias.gulder@ch.tum.de (T.A.M.G); Tel.: +49-89-289-13833. jimhoff@geomar.de (J.F.I.); Tel.: +49-431-600-4450

Supporting Information

[Spectroscopic data of asperentin B (**2**)]

- ^{1}H NMR data of asperentin B (**1**)

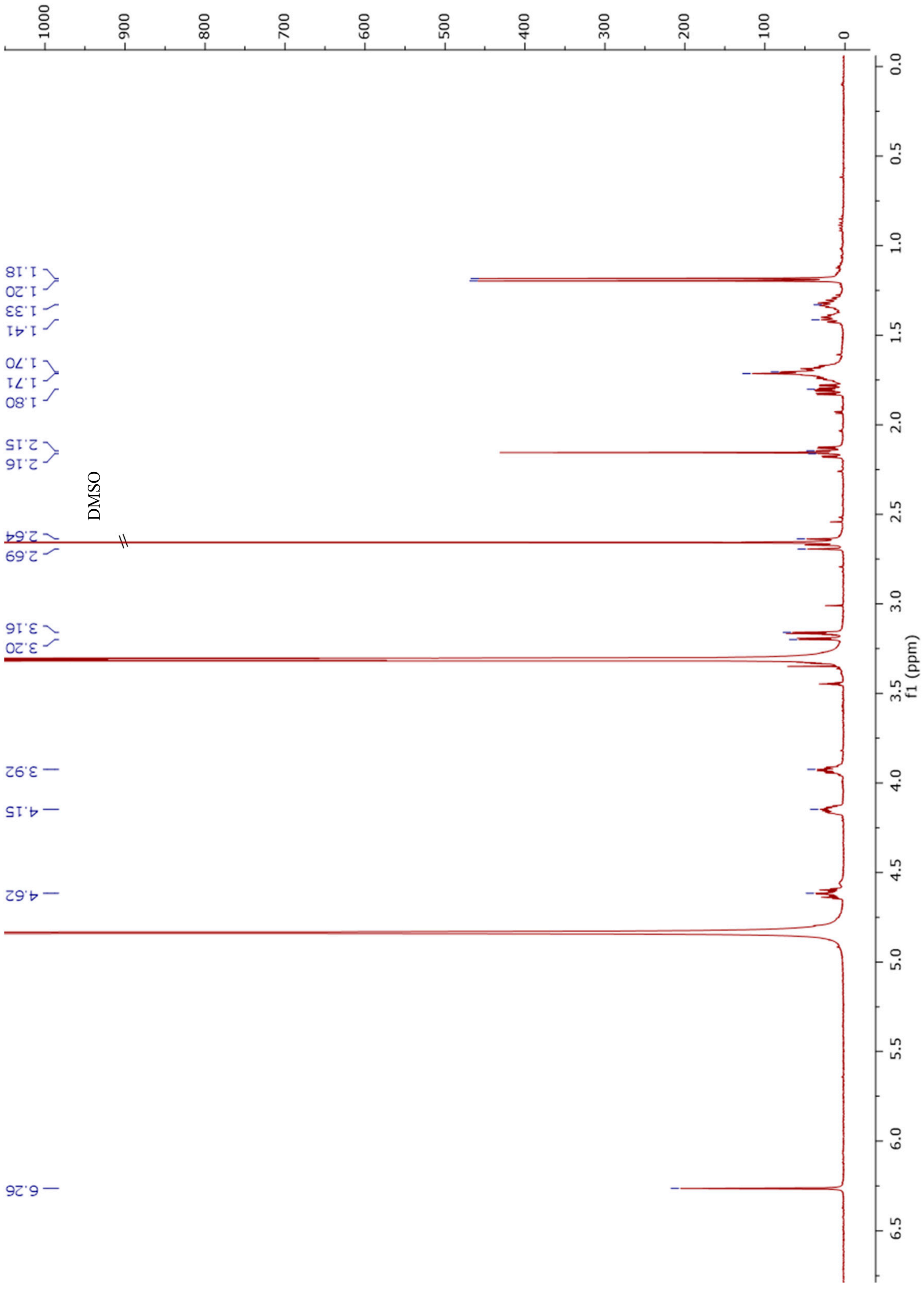

- ^{1}H-^{1}H-COSY NMR data of asperentin B (**1**)

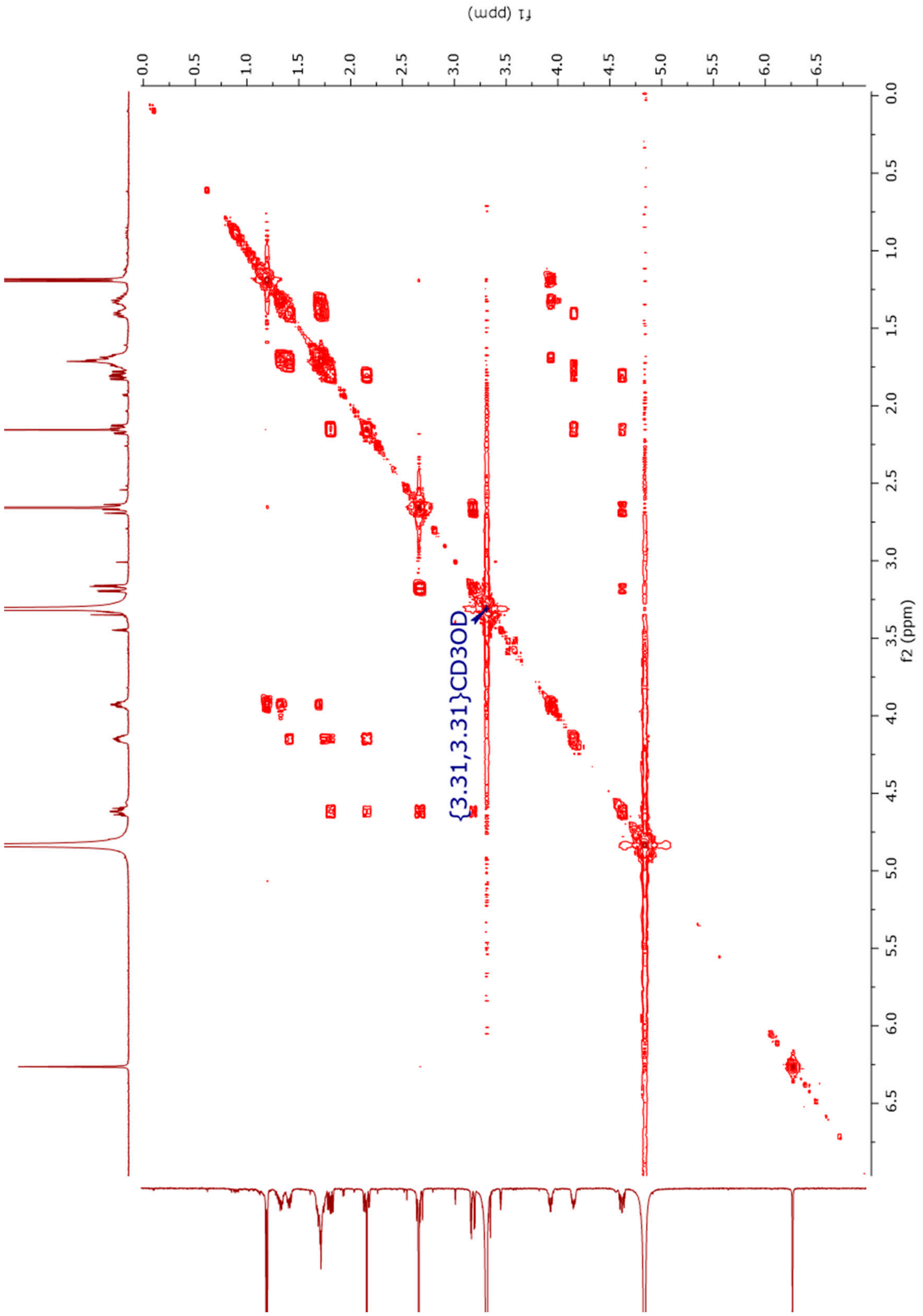

- HSQC NMR data of asperentin B (**1**)

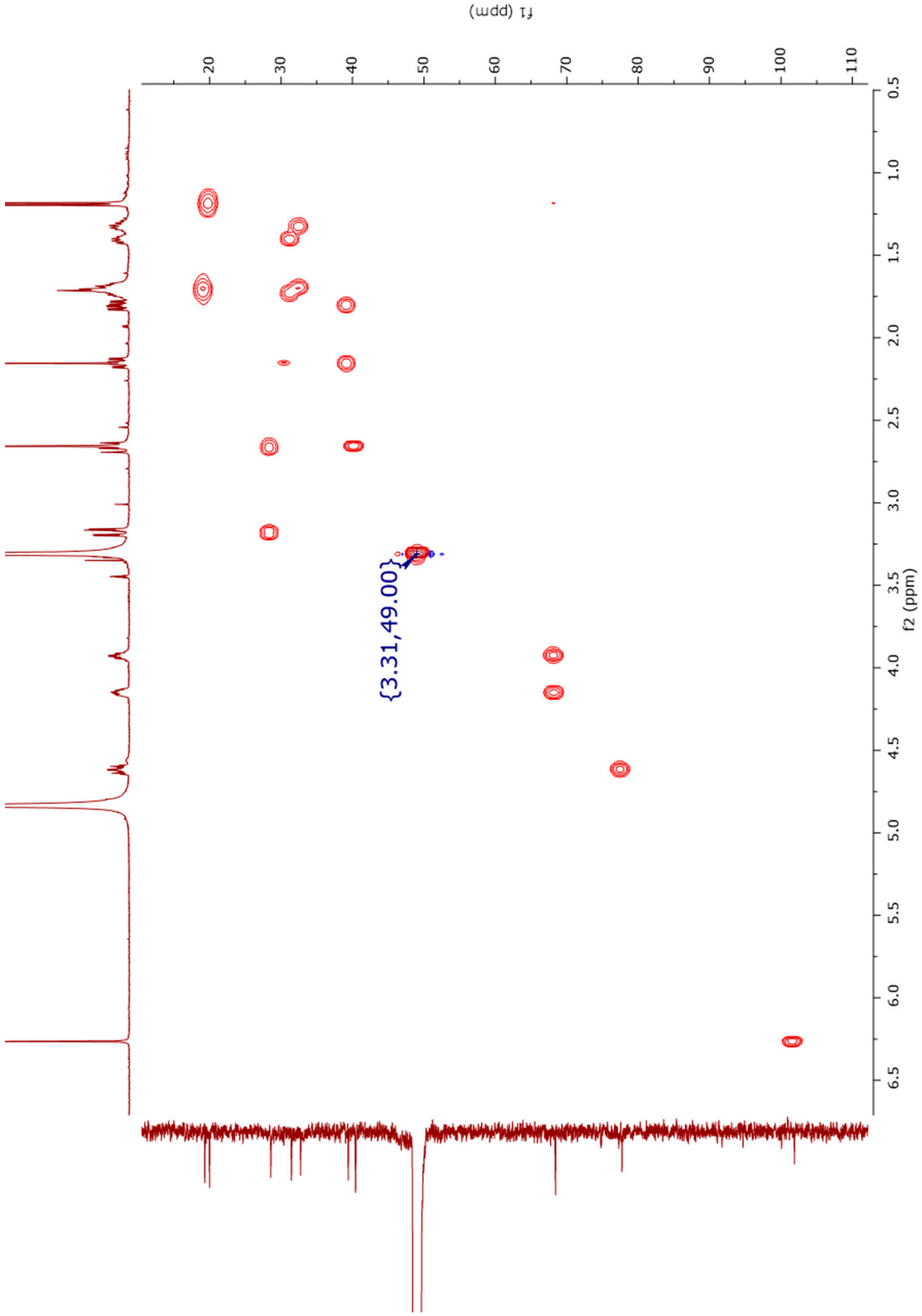

- HMBC NMR data of asperentin B (**1**)

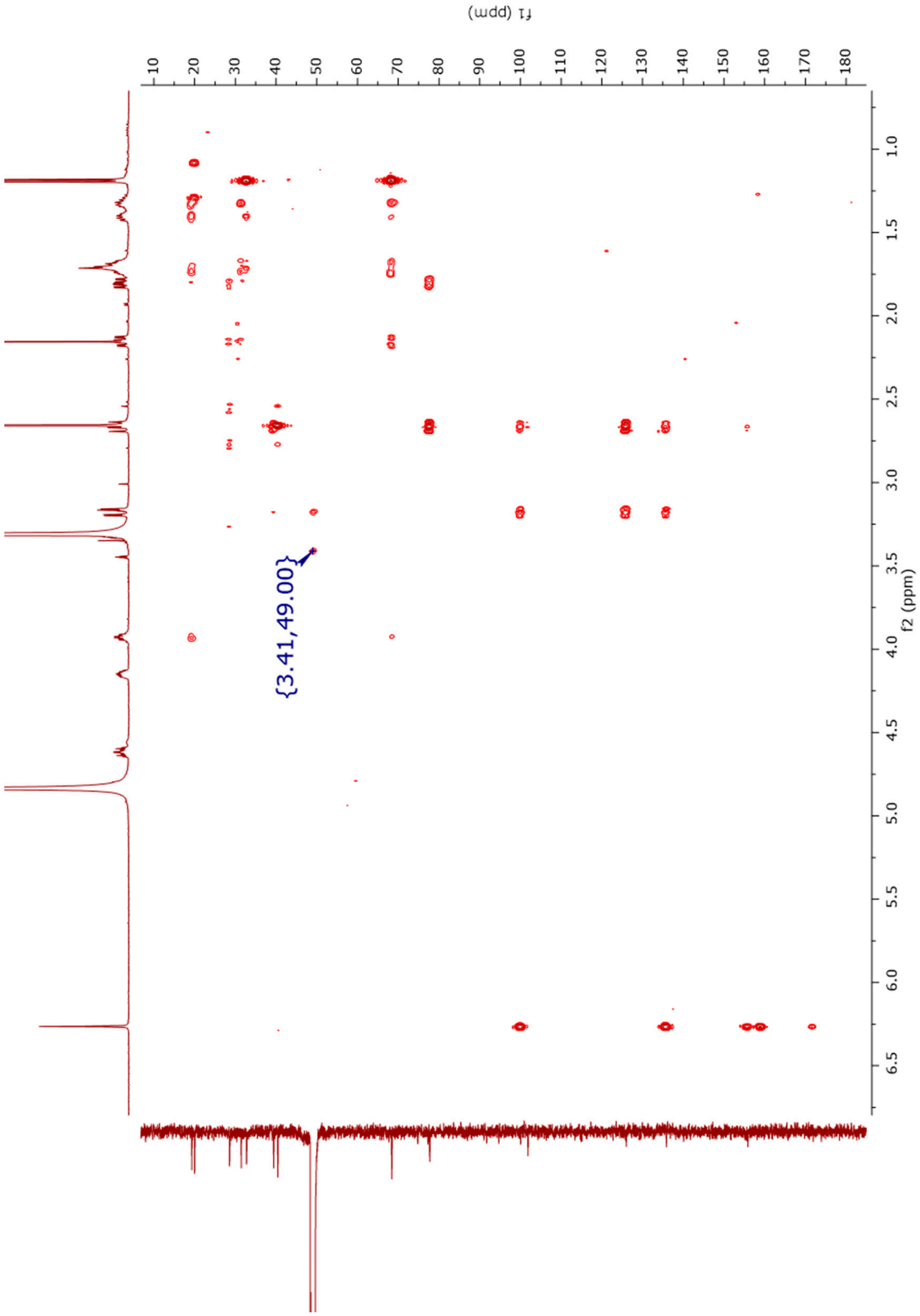

- ^{13}C NMR data of asperentin B (**1**)

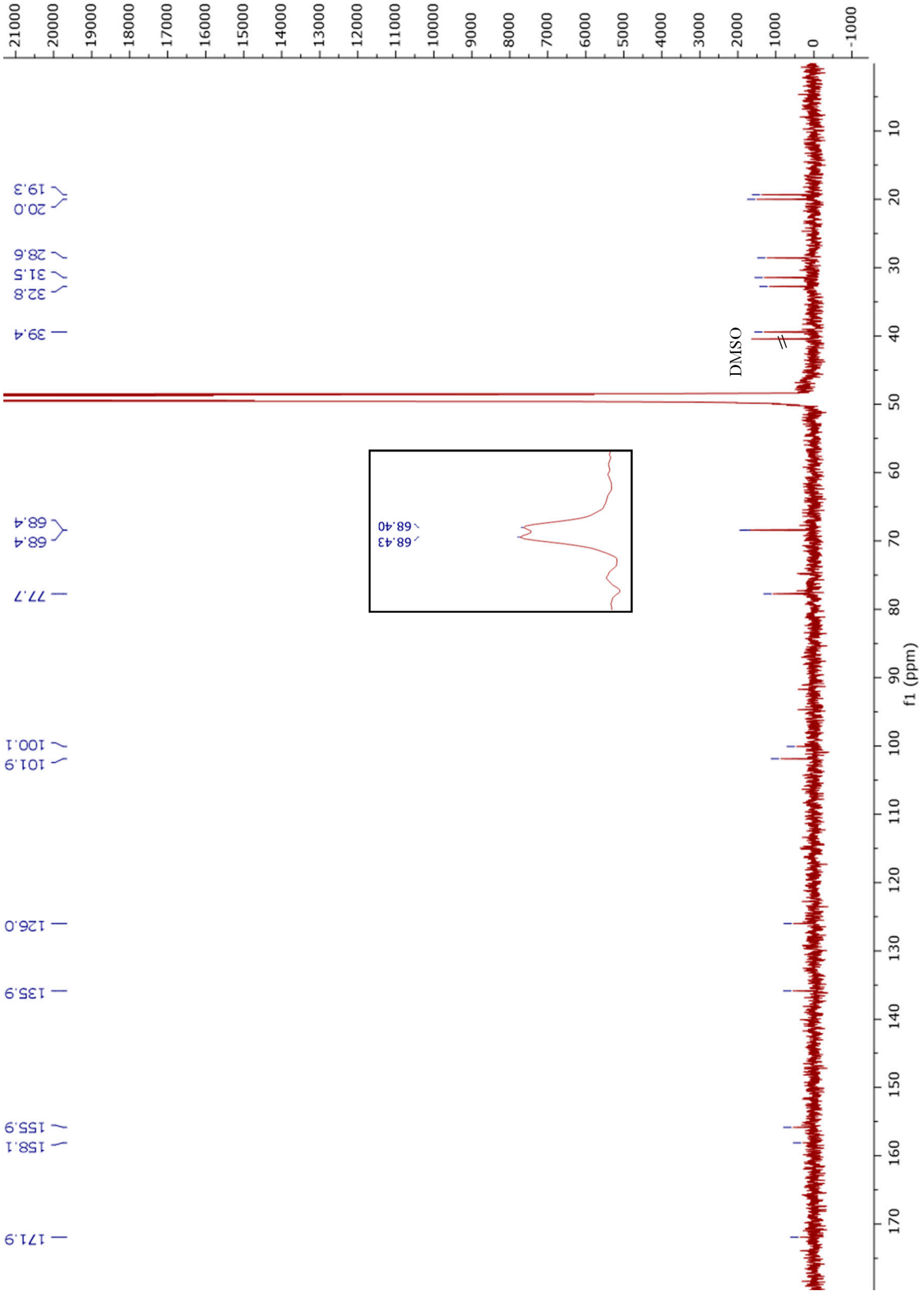

Supplement: Supplementary file 1 [file marinedrugs-15-00191-s001.pdf]
